# Micro- and Macro-Level Time Associations: How Daily Life Settles Into Longer-Term Profiles

**DOI:** 10.1093/geroni/igaa057.2051

**Published:** 2020-12-16

**Authors:** Raquael Joiner, Niccole Nelson, Stacey Scott

## Abstract

Over a decade ago, Ram and Gerstorf (2009) proposed a descriptive framework to unite the study of intraindividual variability, operating at the micro-level timescale (e.g., minutes, days), and intraindividual change, operating at the macro-level time scale (e.g., years, decades). Since this proposal, several aging theories have incorporated a micro-level time component in their conceptualizations of longer-term aging processes. Furthermore, technological advancements have eased difficulties associated with data-collection at micro-level timescales, leading to an upsurgence of empirical investigations of dynamic characteristics and dynamic processes. This session presents theoretical, quantitative, and qualitative research aimed at better understanding the associations between micro- and macro-level time. More specifically, 1) Nelson et al. present their novel theoretical framework linking micro-level time emotion regulatory processes to intraindividual trajectories of cognitive functioning, 2) Joiner and colleagues present a quantitative study assessing the association between daily emotion-dynamics and yearly trajectories of depressive symptomatology, 3) Bergeman et al. present a quantitative study of daily risk and resilience in relation to trajectories of health and well-being, and 4) Bouklas and colleagues present a qualitative study linking individuals’ daily routines and behaviors to their general life outlooks. The quantitative and qualitative studies are based on available data from the The Notre Dame Study of Health & Well-Being, a 10-year, nested-longitudinal study that incorporates yearly questionnaires, five 56-day measurement bursts, and interview data. Discussant Stacey Scott will synthesize the presentations with Ram and Gerstorf’s framework and encourage researchers to integrate shorter- and longer-term timescales into their theoretical and empirical work on aging.

